# Generation of the RCPCMi014-A and RCPCMi014-B lines from T-lymphocytes of a healthy donor and verification of their monoclonal origin by TCR V(D)J rearrangement analysis

**DOI:** 10.18699/vjgb-26-40

**Published:** 2026-05

**Authors:** D.K. Sherman, M.E. Bogomiakova, E.A. Kastueva, I.V. Zvyagin, V.K. Ruppel, E.V. Barsova, A.Y. Gorbachev, N.A. Kulemin, A.A. Barinova, E.A. Zerkalenkova, A.N. Bogomazova, M.A. Lagarkova

**Affiliations:** Lopukhin Federal Research and Clinical Center of Physical-Chemical Medicine of Federal Medical Biological Agency, Moscow, Russia; Lopukhin Federal Research and Clinical Center of Physical-Chemical Medicine of Federal Medical Biological Agency, Moscow, Russia Center for Precision Genome Editing and Genetic Technologies for Biomedicine, Lopukhin Federal Research and Clinical Center of Physical-Chemical Medicine of Federal Medical Biological Agency, Moscow, Russia; Lopukhin Federal Research and Clinical Center of Physical-Chemical Medicine of Federal Medical Biological Agency, Moscow, Russia; Shemyakin−Ovchinnikov Institute of Bioorganic Chemistry, Russian Academy of Sciences, Moscow, Russia; Shemyakin−Ovchinnikov Institute of Bioorganic Chemistry, Russian Academy of Sciences, Moscow, Russia LLC “MiLaboratori”, Moscow, Russia; Shemyakin−Ovchinnikov Institute of Bioorganic Chemistry, Russian Academy of Sciences, Moscow, Russia LLC “MiLaboratori”, Moscow, Russia; Lopukhin Federal Research and Clinical Center of Physical-Chemical Medicine of Federal Medical Biological Agency, Moscow, Russia; Lopukhin Federal Research and Clinical Center of Physical-Chemical Medicine of Federal Medical Biological Agency, Moscow, Russia; Lopukhin Federal Research and Clinical Center of Physical-Chemical Medicine of Federal Medical Biological Agency, Moscow, Russia; Dmitry Rogachev National Medical Research Center of Pediatric Hematology, Oncology and Immunology, Moscow, Russia Pirogov Russian National Research Medical University, Moscow, Russia; Lopukhin Federal Research and Clinical Center of Physical-Chemical Medicine of Federal Medical Biological Agency, Moscow, Russia Center for Precision Genome Editing and Genetic Technologies for Biomedicine, Lopukhin Federal Research and Clinical Center of Physical-Chemical Medicine of Federal Medical Biological Agency, Moscow, Russia; Lopukhin Federal Research and Clinical Center of Physical-Chemical Medicine of Federal Medical Biological Agency, Moscow, Russia

**Keywords:** induced pluripotent stem cells, T-lymphocytes, reprogramming, T-cell receptor, cell clone, V(D)J recombination, definitive endoderm, индуцированные плюрипотентные стволовые клетки, Т-лимфоциты, репрограммирование, T-клеточный рецептор, клон клеток, V(D)J-рекомбинация, дефинитивная эндодерма

## Abstract

The generation of induced pluripotent stem cells (iPSCs) from peripheral blood T-lymphocytes offers a promising alternative to skin fibroblasts. This approach combines minimally invasive sample collection with rapid isolation of enriched target cell population and provides a lower baseline mutational burden, as T-lymphocytes are shielded from chronic ultraviolet radiation – a major driver of somatic mutations in skin fibroblasts. Objective verification of monoclonality of the resulting iPSC lines is possible due to V(D)J rearrangements in T-cell receptor (TCR) genes, which are formed as a result of somatic recombination in the thymus during the natural maturation of T-lymphocytes. These rearrangements remain unchanged during reprogramming and serve as a unique marker, allowing for reliable identification of monoclonal lines and exclusion of contaminated or polyclonal lines. Reliable verification of the clonal origin of iPSCs can be crucial in experiments that place high demands on genetic homogeneity. This work is dedicated to the generation and comprehensive characterization of monoclonal iPSC lines derived from CD3+ T-lymphocytes of a healthy donor using episomal reprogramming. The resulting lines, RCPCMi014-A (PBM022E5) and RCPCMi014-B (PBM022E7), meet the accepted quality criteria for iPSCs: they demonstrate expression of key pluripotency markers (OCT4, SOX2, SSEA-4, TRA-1-81), the ability to differentiate into derivatives of all three germ layers, and possess a normal karyotype. Both lines showed a high-efficiency capacity to differentiate into definitive endoderm, as assessed by CXCR4 expression, highlighting their potential for developing protocols to generate pancreatic β-cells. The obtained iPSC lines can be used for fundamental research into the mechanisms of pluripotency and differentiation, as well as for creating isogenic iPSC lines with introduced mutations for modeling rare hereditary diseases.

## Abstract (ru)

Получение индуцированных плюрипотентных стволовых клеток из Т-лимфоцитов периферической крови представляет собой перспективную альтернативу использованию фибробластов кожи. Этот подход сочетает минимально инвазивный забор биоматериала совместно с быстрым получением обогащенной популяции целевых клеток. Дополнительным преимуществом является относительно низкий мутационный груз T-лимфоцитов по сравнению с традиционным источником соматических клеток для репрограммирования – фибробластами кожи, ввиду того что Т-лимфоциты защищены от хронического воздействия ультрафиолетового излучения – одного из ключевых факторов накопления соматических мутаций. Объективная верификация моноклональности полученных линий индуцированных плюрипотентных стволовых клеток возможна благодаря наличию V(D)J-перестроек генов Т-клеточного рецептора, формирующихся в результате соматической рекомбинации в тимусе в процессе естественного созревания Т-лимфоцитов. Эти перестройки остаются неизменными при репрограммировании и служат уникальным маркером, позволяющим достоверно идентифицировать моноклональные линии и исключать контаминированные линии или линии поликлонального происхождения. Надежная верификация клонального происхождения индуцированных плюрипотентных стволовых клеток может быть важной в экспериментах, предъявляющих высокие требования к генетической однородности. Данная работа посвящена созданию и комплексной характеристике моноклональных линий индуцированных плюрипотентных стволовых клеток, полученных из CD3+ Т-лимфоцитов здорового донора методом эписомного репрограммирования. Полученные линии RCPCMi014-A (PBM022E5) и RCPCMi014-B (PBM022E7) соответствуют принятым критериям качества для индуцированных плюрипотентных стволовых клеток: они демонстрируют экспрессию ключевых маркеров плюрипотентности (OCT4, SOX2, SSEA-4, TRA-1- 81), способность к дифференцировке в производные всех трех зародышевых листков и обладают нормальным кариотипом. Обе линии показали способность к дифференцировке в дефинитивную эндодерму с высокой эффективностью, оцененной по экспрессии CXCR4, что определяет их перспективность для разработки протоколов генерации β-клеток поджелудочной железы. Полученные линии индуцированных плюрипотентных стволовых клеток могут быть использованы для фундаментальных исследований механизмов плюрипотентности и дифференцировки, а также для создания изогенных линий индуцированных плюрипотентных стволовых клеток с внесенными мутациями при моделировании редких наследственных заболеваний.

## Introduction

The discovery of induced pluripotent stem cells (iPSCs) in 2006 opened up new possibilities in regenerative medicine and biomedical research (Yamanaka, 2020). These cells have the ability to differentiate into all three germ layers and hold great potential for disease modeling in vitro. This allows researchers to study tissue-specific pathological processes at a cellular level (Summers et al., 2024). Typically, iPSC models are created from patients with inherited diseases (Grigor’eva et al., 2023), which helps to replicate individual disease characteristics. Healthy donor lines serve as important reference controls in these studies. They are especially valuable for studying orphan diseases, which have a small patient population, making it difficult to access cellular models directly. In these cases, creating isogenic pairs through editing healthy donor iPSCs is a promising solution (Khomyakova et al., 2023; Fedorenko et al., 2024). It is important to note that iPSCs from healthy donors represent an essential resource for standardizing differentiation protocols, creating “universal” cell products, and studying basic reprogramming mechanisms (Cerneckis et al., 2024).

According to established practice, each morphologically homogeneous iPSC colony is considered to be a clone originating from a single reprogrammed precursor cell (Takahashi et al., 2007). Following this practice, individual colonies are cultured independently and are regarded as distinct cell lines. However, direct confirmation of monoclonality is rarely performed, and the lack of verification methods can lead to polyclonal lines going undetected, especially when colonies form due to the close proximity of cells. Cell line authentication, which includes confirmation of genetic identity and the absence of contamination, is a crucial laboratory procedure for ensuring research reproducibility, minimizing financial losses, and maintaining data reliability (Harbut et al., 2024).

In this context, one of the unique advantages of iPSCs derived from T-lymphocytes is the ability to verify monoclonal origin through analysis of genomic rearrangements that occur during V(D)J recombination during the formation of T-cell receptor (TCR) gene chains (Kishino et al., 2014). These clone-specific rearrangements, which are preserved after reprogramming, serve as a molecular “barcode” for the original T-cell. Modern approaches to V(D)J analysis, such as multiplex PCR (Seki et al., 2010) and targeted highthroughput sequencing (Nishimura et al., 2013), enable direct detection of polyclonal origins, which is crucial for ensuring the reproducibility and reliability of biomedical research results. The combination of this objective clonality verification with minimally invasive biomaterial collection makes T-lymphocyte-derived iPSCs an attractive strategy for both basic research and disease modeling applications.

In this study, iPSC lines were generated from healthy donor T-lymphocytes using episomal reprogramming. For the first time, polyclonal populations were excluded by analyzing V(D)J rearrangements at three genomic loci, allowing for reliable identification of monoclonal lines. The iPSC lines RCPCMi014-A (PBM022E5) and RCPCMi014-B (PBM022E7) with confirmed monoclonal origin demonstrated full compliance with quality criteria, including maintenance of a normal karyotype, expression of key markers (OCT4, SOX2, SSEA-4, TRA-1-81), and the capacity to differentiate into derivatives of all three germ layers. Specifically, they showed high efficiency in directed differentiation into definitive endoderm.

## Materials and methods

**Ethical considerations and collection of biological materials.** The study was approved by the Research Ethics Committee of the Lopukhin Federal Research and Clinical Center of Physical-Chemical Medicine of Federal Medical Biological Agency, Protocol No. 1, dated June 1, 2021. The biological material (peripheral venous blood) was obtained from a 35-year-old male volunteer donor. Blood sampling was performed by qualified medical staff of the clinic at the Lopukhin FRCC PCM Russia, in accordance with all standard medical protocols. Prior to the procedure, the donor was informed about the research objectives and provided voluntary informed consent for the use of his biological material for research purposes.

**Exome sequencing and bioinformatic analysis.**Primary bioinformatic processing included quality control of raw reads using FastQC, with subsequent filtering of low-quality reads and adapter sequences. Read alignment to the human reference genome GRCh37/hg19 was performed using the BWA-MEM algorithm. Alignment quality and enrichment efficiency were assessed using SAMtools, mosdepth, and Picard Tools. Detection of single nucleotide variants and small insertions/deletions (SNV/InDel) was conducted with the DeepVariant software package. Genomic DNA was extracted from frozen whole blood samples using the ExtractDNA Blood & Cells kit (Evrogen). The sequencing library was prepared with the MGIEasy Universal DNA Library Prep Set (MGI), and exome regions were enriched using the VAHTS Target Capture Core Exome Panel (Vazyme), following the manufacturer’s protocols. The resulting library underwent paired-end sequencing on the MGISEQ-2000 platform (MGI), achieving coverage of ~80x.

Isolation and activation of T-lymphocytes. Peripheral blood mononuclear cells (PBMCs) were isolated from venous blood by density gradient centrifugation using NycoPrep ™ reagent (Axis-Shield) according to the standard protocol (Grigor’eva et al., 2024). CD3+ T-cells were subsequently isolated from PBMCs using magneticactivated cell sorting with the Human CD3+ Cell Separation Kit (RWD), following the manufacturer’s protocol. T-lymphocyte activation was performed using a modified two-step protocol (Maslennikova et al., 2022). During the first step, the cells were incubated with Dynabeads™ Human T-Activator CD3/CD28 magnetic particles (Gibco). After 24 hours, beads were removed using a magnetic stand, and the cells were resuspended in T-cell medium consisting of RPMI-1640 (PanEco) supplemented with 10 % heat-inactivated HI-FBS (BioFroxx) and 100 IU/mL IL-2 (Biotech NPK) at a concentration of 0.5×106 cells/mL for an additional 24 hours of culture.

**Reprogramming of T-cells and iPSC culture.** T-cell reprogramming was performed via transfection with episomal vectors (Okita et al., 2013) expressing reprogramming factors SOX2, OCT4, KLF4, L-MYC, LIN28, a dominant-negative form of mouse p53 (mp53DD), and the episomal maintenance factor EBNA1 (Addgene #41813-14, 41855-57). Transfection was carried out using the Neon Transfection System (ThermoFisher Scientific) with standard parameters: 1,650 V, 10 ms, 3 pulses. The pCE-GFP plasmid (Addgene #41858) served as a transfection efficiency control. Twenty-four hours postelectroporation, the number of viable, DAPI-negative activated blast cells – characterized by increased size (FSC) and granularity (SSC) – was quantified using a NovoCyte flow cytometer (ACEA Biosciences). Cell suspensions were plated on 35-mm Petri dishes pre-coated with 1:50 diluted Matrigel (Corning) at a density of 10–12 × 103 viable blasts/cm2 in T-cell medium. The following day, an equal volume of reprogramming medium was added, consisting of ReproTeSR medium (Stemcell Technologies), 100 ng/ mLFGF2 (produced in-house), 10 μM ROCK inhibitor Y-27632, 0.5 μM PD0325901, and 2 μM SB431542 (all from DC Chemicals). Reprogramming medium was subsequently replaced every other day. On day 16, cells were treated with modified iPSC medium composed of a 4:1 mixture of GibberS-8 (PanEco) and mTeSR1 (Stemcell Technologies), with daily medium changes. On day 26, colonies were mechanically isolated using 0.1 % dispase solution (Invitrogen). The resulting iPSC clones were maintained under feeder-free conditions according to a previously described protocol (Goliusova et al., 2025).

**Analysis of transgene integration by real-time qPCR.** To assess potential integration of episomal vectors into the genome, iPSC lines were cultured for at least 10 passages. Genomic DNA was subsequently extracted using the M-Sorb kit (Syntol). Transgene integration analysis was performed by real-time quantitative PCR on a CFX96 Touch Real-Time PCR Detection System (BioRad) using 5X qPCRmix-HS SYBR (Evrogen) and specific primers (Supplementary Table S1)1. The amplification protocol consisted of the following steps: initial denaturation at 95 °C for 4 min; 40 cycles of 95 °C for 10 s and 60 °C for 40 s (with fluorescence detection); and a final melting curve analysis: 95 °C – 5 s, 65 °C – 5 s (with continuous fluorescence acquisition), 95 °C – 50 s).

Supplementary Materials are available in the online version of the paper: https://vavilov.elpub.ru/jour/manager/files/Suppl_Sher_Engl_30_3.pdf


**Analysis of V(D)J rearrangement sequences.** To determine the clonal composition of iPSC lines, we analyzed sequences of three genomic loci formed through V(D) J recombination that determine the mature α/δ-, β- and γ-chain TCR genes. High-throughput sequencing libraries were prepared from 42 ng of genomic DNA (equivalent to ~6,500 diploid genomes) using the Human IG/TCR DNA Multiplex Kit 7G (MyLaboratory) according to the manufacturer’s protocol. Sequencing was performed on the Illumina MiSeq platform in paired-end mode with 150 bp read length. A minimum of 90,000 paired-end reads were obtained for each sample.

Bioinformatic analysis was conducted using the MiXCR software package (https://mixcr.com). A minimum of 83 % of reads in each sample were successfully identified as containing sequences unambiguously attributable to one of the investigated loci through alignment with reference sequences of the corresponding loci using MiXCR software. To determine clonality, we examined the distribution of read counts and predicted functionality of identified V(D) J rearrangement sequences in each of the three loci. Rearrangements supported by at least 100 independent reads were included in the analysis

**Mycoplasma screening.** The absence of mycoplasma contamination was confirmed by PCR using primers targeting the 16S ribosomal RNA gene (Table S1), as previously described (Goliusova et al., 2024).

**RNA isolation and RT-PCR.**Total RNA was isolated from 1 million cells using Extract RNA reagent (Evrogen) according to the manufacturer’s protocol. Reverse transcription was performed using the MMLV RT kit (Evrogen) and random decanucleotide primers. RT-PCR was conducted using specific primers (Table S1). The amplification protocol was: 95 °C for 3 min; 32 cycles of 95 °C for 15 s, 60 °C for 15 s, 72 °C for 30 s; final elongation: 72 °C for 5 min.

**Karyotyping.** Karyotyping was performed according to a previously described method at a resolution of 400 bands (Goliusova et al., 2024).

**STR analysis for cell line authentication. **STR profiling was conducted using the commercial COrDIS Plus kit (Gordiz), which analyzes 19 autosomal loci and the sex marker Amelogenin

**Analysis of pluripotency marker expression by flow cytometry.**Flow cytometry was performed according to previously described methodology (Khomyakova et al., 2023). Antibodies used in the study are listed in Table S2.

**Immunocytochemical staining.** Immunocytochemical staining was carried out following a previously published protocol (Fedorenko et al., 2024). Antibodies used in the study are listed in Table S2. Visualization was performed using an Olympus IX53F inverted fluorescence microscope. Image processing was conducted using GIMP-2.10 software.

**Functional assessment of pluripotency by spontaneous differentiation.** To confirm the pluripotent status of iPSCs, a spontaneous differentiation test was performed to assess their ability to form derivatives of the three germ layers, following previously described protocol (Goliusova et al., 2024). After 15–20 days of adherent culture, embryoid bodies were fixed with 4 % PFA and stained for specific markers of the three germ layers (Table S2).

**Differentiation into definitive endoderm.** Differentiation into definitive endoderm was performed using the STEMdiff ™ Definitive Endoderm Kit (STEMCELL Technologies) according to the manufacturer’s protocol. Differentiation efficiency was evaluated based on the expression of the CXCR4 marker using antibodies listed in Table S2.

## Results and discussion

**Fig. 1.**
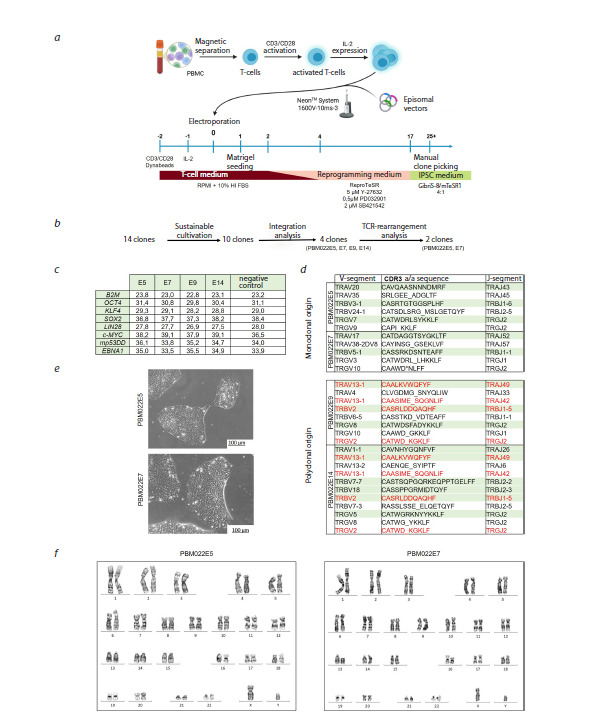
T-cell reprogramming: a – schematic interpretation of T-cell activation and reprogramming; b – strategy for iPSC clone selection and validation; c – transgene integration analysis by Ct values: E5, E7, E9, E14 represent lines PBM022E5, PBM022E7, PBM022E9, PBM022E14, respectively; negative control: iPSC line RCPCMi007-A-1 (Bogomiakova et al., 2021). Comparable Ct values with the negative control indicate the absence of residual transgenes in the investigated clones; d – analysis of V(D)J rearrangement sequences: underline indicates frameshift disruption; green highlights, presumably functional chains without frameshift disruption; red highlights, V(D)J rearrangement variants shared between clones PBM022E9 and PBM022E14; e – morphology of the PBM022E5 and PBM022E7 lines; f – karyotype of the PBM022E5 and PBM022E7 lines.

This study focuses on the generation and detailed characterization of iPSC lines derived from T-lymphocytes of a healthy donor. Whole-exome sequencing of the donor revealed no pathogenic or likely pathogenic variants in genes associated with oncological diseases, according to the PanelApp database (Martin et al., 2019) and ACMG guidelines (Richards et al., 2015). The reprogramming scheme is presented in Figure 1a. CD3+ T-lymphocytes isolated from peripheral blood were activated using CD3/ CD28 DynabeadsTM and transfected with a cocktail of episomal plasmids encoding OCT4, SOX2, KLF4, c-MYC, LIN28, EBNA1, and mp53DD. Twenty-four hours posttransfection, cells were plated on Matrigel-coated dishes and cultured in medium supplemented with a combination of small molecules: Y-27632 (ROCK inhibitor), SB431542 (TGF-β inhibitor), and PD0325901 (MEK inhibitor), which enhances cell survival and reprogramming efficiency (Watanabe et al., 2019). Three weeks after transfection, the formation of compact colonies morphologically resembling embryonic stem cell colonies was observed.

The overall strategy for iPSC clone selection and validation is presented in Fig. 1b. Episomal reprogramming of T-lymphocytes yielded 14 primary iPSC colonies (PBM022E1-E14) selected based on morphological criteria. Each colony was isolated and cultured as a separate cell line, consistent with standard practice in iPSC generation (Takahashi et al., 2007). Theoretically, morphologically homogeneous colonies could form from closely located reprogrammed cells, potentially resulting in polyclonal lines. While some studies demonstrate that such lines can maintain functional stability (Willmann et al., 2013), others show that polyclonality may manifest as differences in differentiation capacity (Yun et al., 2025) and proliferation rate (Mills et al., 2013), which could reduce experimental reproducibility.

Among the 14 primary iPSC clones obtained through reprogramming, 10 demonstrated stable cultivation for over 80 days (minimum 10 passages) and were selected for further functional characterization. Transgene integration analysis by qPCR revealed that four clones (PBM022E5, PBM022E7, PBM022E9, PBM022E14) completely lost episomal vectors (Fig. 1c). The iPSC line RCPCMi007- A-1, generated using non-integrating Sendai virus vectors (Bogomiakova et al., 2021), served as a negative control free of transgene integrations. The B2M gene, encoding beta-2-microglobulin protein, was used as a reference single-copy gene located on an autosomal chromosome. These findings align with literature reports on episomal plasmid elimination in other iPSC lines generated using this method (Grigor’eva et al., 2024; Podvysotskaya et al., 2025).

Compared to other somatic cells, mature T-lymphocytes enable objective confirmation of the monoclonality of derived iPSCs through analysis of genomic loci sequences generated by V(D)J recombination during T-cell maturation in the thymus (Alt et al., 1992). The random combina- tion of V, D, and J segments, along with nucleotide insertions and deletions at their junctions, creates a CDR3 region with a sequence unique to each original T-cell precursor. This sequence is inherited by all cellular descendants and persists after reprogramming, enabling assessment of the clonal diversity in iPSC lines derived from mature T-lymphocytes.

Given the predominance of αβT-cells in the peripheral blood of a healthy adult donor, the obtained iPSC clones most likely originated from mature T-lymphocytes with functional TCR α- and β-chain genes (Kreslavsky et al., 2008, 2010). The criterion for monoclonality was defined as the presence of at least one productive V(D)J rearrangement pair in the TRA and TRB loci. The presence of multiple productive rearrangements and/or a total of more than two rearrangements in either locus would indicate polyclonal origin. Rearrangements in the TRG locus would not affect the interpretation of clonality based on the TRA and TRB loci, provided no rearrangements were detected in the δ-chain gene (located within the TRAD locus) and the total number of TRG rearrangements did not exceed two (Sherwood et al., 2011; Mahe et al., 2018).

V(D)J repertoire analysis confirmed that all clones originated from αβT-cells, as productive rearrangements of the TCR δ-chain gene were absent. Two clones – PBM022E5 and PBM022E7 – exhibited a rearrangement pattern typical of monoclonal origin, characterized by one productive sequence each for the TCR α- and β-chain genes (Fig. 1d, highlighted in green). This profile allows these lines to be considered with high confidence as descendants of individual parental T-cells. In contrast, clone PBM022E14 was classified as polyclonal due to the presence of three productive β-chain and two α-chain TCR rearrangements (Fig. 1d). In total, four V(D)J rearrangements were identified in both the TRA and TRB loci of this clone (Table S3). Clone PBM022E9 displayed an asymmetric profile: one productive β-chain sequence was detected alongside three α-chain variants, suggesting potential polyclonal origin. Analysis of shared V(D)J rearrangements across all four investigated lines revealed identical TCR α- and β-chain sequences in clones PBM022E9 and PBM022E14 (Fig. 1d and Table S3, highlighted in red), indicating possible technical contamination.

Thus, analysis of genomic regions formed during V(D) J recombination in T-lymphocyte maturation represents an effective tool for verifying the clonality of iPSCs derived from mature T-lymphocytes. The unique CDR3 region sequence, generated through random combination of V, D, and J segments, serves as a reliable “genetic barcode” for each original cell, providing irrefutable evidence of monoclonal origin. The stability of this marker, which remains unchanged after reprogramming, enables its use for long-term clonal identification, including in differentiated derivatives. This approach becomes particularly valuable when working with unique models, such as iPSCs from monozygotic twins, where traditional DNA polymorphismbased genotyping methods lose informativeness due to the near-complete genetic identity of donors (Vlasov et al., 2021). Additionally, the method exhibits high sensitivity to contamination, enabling detection of cross-contamination between lines through the presence of identical V(D) J sequences.

However, the method has certain limitations. Firstly, it is applicable only to T-cell origins and cannot be used for iPSCs derived from other somatic cell types, such as fibroblasts, which lack analogous cell-specific markers. Secondly, result interpretation requires establishing clear threshold values (in our work – 100 reads) to distinguish technical errors from true polyclonality, as well as specialized sequencing equipment and bioinformatics expertise for data analysis. Despite these limitations, V(D)J profiling represents a powerful tool for standardizing cellular models and enhancing research reproducibility in cell biology.

**Fig. 2.**
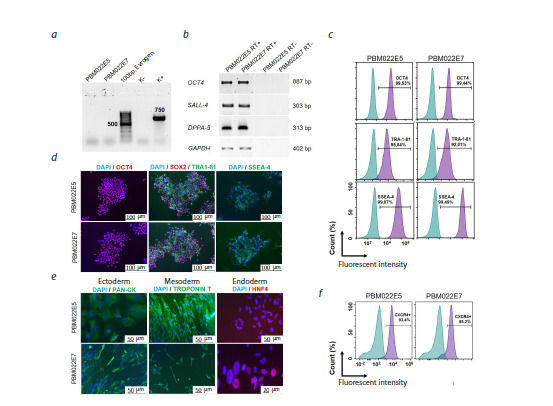
Characterization of iPSC lines RCPCMi014-A (PBM022E5) and RCPCMi014-B (PBM022E7): a – PCR analysis for mycoplasma contamination; b – RT-PCR analysis of pluripotency marker expression; c – flow cytometry analysis of pluripotency markers; d – immunocytochemical staining of pluripotency markers: OCT4 (red), SOX2 (red), TRA-1-81 (green), SSEA-4 (green), nuclei stained with DAPI (blue); e – immunocytochemical staining of three germ layer markers: ectoderm PAN-CK (green), mesoderm TROPONIN T (green), endoderm HNF-4 (red); f – flow cytometry analysis of the definitive endoderm early marker CXCR4.

The obtained iPSC lines PBM022E5 (RCPCMi014-A) and PBM022E7 (RCPCMi014-B) were registered in the Human Pluripotent Stem Cell Registry (hPSCreg, https:// hpscreg.eu). Information about these cell lines can be found at the following links: https://hpscreg.eu/cell-line/ RCPCMi014-A and https://hpscreg.eu/cell-line/RCPCMi014- B. Both lines demonstrated typical pluripotent stem cell morphology (Fig. 1e), normal karyotype (46, XY) (Fig. 1f ), and confirmed genetic identity with the donor through STR profiling (data available from the authors upon request). All lines were tested for mycoplasma contamination, confirmed by negative PCR results (Fig. 2a). Expression of key pluripotency markers was confirmed by several methods. Immunocytochemical analysis revealed nuclear expression of OCT4 and SOX2, as well as surface expression of TRA-1-81 and SSEA-4 (Fig. 2d). RT-PCR results showed high expression levels of the OCT4, SALL4, and DPPA5 genes (Fig. 2b). Flow cytometry data confirmed high percentages of positive cells: over 99 % expressed OCT4, over 99 % expressed SSEA-4, and over 92 % expressed TRA-1-81 (Fig. 2c). The capacity for differentiation into derivatives of all three germ layers was assessed through spontaneous in vitro differentiation. Expression of pan-cytokeratin confirmed ectoderm formation, troponin T confirmed it for mesoderm (including cardiomyocytes), and HNF4α confirmed it for endoderm (Fig. 2d).

The next aspect of characterization was the assessment of the lines’ capacity to differentiate into definitive endoderm – a critical step for subsequent generation of pancreatic β-cells. Our data reveal significant variability in differentiation potential among different lines: only onethird of iPSC lines demonstrate high efficiency in definitive endoderm formation (Fig. S1). Flow cytometry analysis showed that lines PBM022E5 and PBM022E7 exhibit high and stable capacity for definitive endoderm differentiation, as evidenced by CXCR4 marker expression in over 85 % of cells (Fig. 2f ). These results confirm the potential utility of these lines for developing and optimizing protocols for directed differentiation into insulin-producing cells. Cell line passports for RCPCMi014-A (PBM022E5) and RCPCMi014-B (PBM022E7) are presented in the Table

## Conclusion

In this study, monoclonal iPSC lines reprogrammed from T-lymphocytes of a healthy donor were generated and characterized. Analysis of TCR gene V(D)J rearrangements provided reliable verification of the lines’ monoclonal origin, which is critically important for models requiring high genetic homogeneity. The obtained lines RCPCMi014-A (PBM022E5) and RCPCMi014-B (PBM022E7) were shown to meet all quality criteria for iPSCs and demonstrate high efficiency in differentiating into definitive endoderm, making them promising for the generation of pancreatic β-cells. The established cell lines can serve as reliable reference models both for fundamental research into the mechanisms of pluripotency and differentiation and for applied tasks, including the creation of isogenic pairs for modeling rare and other diseases, as well as for optimizing methodological approaches in regenerative medicine.

## Conflict of interest

The authors declare no conflict of interest.
